# Healthcare reform in the United States and China: pharmaceutical market implications

**DOI:** 10.1186/2052-3211-7-9

**Published:** 2014-07-14

**Authors:** Arthur Daemmrich, Ansuman Mohanty

**Affiliations:** Department of History and Philosophy of Science, University of Kansas School of Medicine, Kansas City, 66160 USA; Life Sciences Consulting, TATA Consultancy Services, Nariman Point, Mumbai, 400021 India

**Keywords:** Affordable care act, Drug prices, Health insurance, Health policy, Healthcare system, Pharmaceutical industry

## Abstract

**Objectives:**

The United States and China are broadening health insurance coverage and increasing spending on pharmaceuticals, in contrast to other major economies that are reducing health spending and implementing a variety of drug price controls. This article analyzes the implications of health system reforms in the United States and China for national pharmaceutical markets. It follows a historical institutionalist approach that identifies path dependency in the design and operation of national health systems. On that basis, we estimate prescription sales for 2015 and 2020, analyze the sustainability of free-market pricing for drugs in the two countries, and assess future competitive dynamics in the pharmaceutical sector.

**Methods:**

The institutional trajectories of health system reform and insurance coverage were studied for the United States and China. Next, data were collected from government, industry, and analyst reports on total healthcare spending and prescription drug expenditure by insurance status (in the United States) and by site of care (in China). Simple quantitative models were developed to estimate future drug spending based on insurance coverage, treatment locations, and health spending as a percentage of GDP.

**Results:**

Both countries will see rising total pharmaceutical spending and will be the two largest country markets for prescription drugs through at least 2020. In dollar terms, the U.S. pharmaceutical market will be over $440 billion in 2015 and $700 billion in 2020; China’s prescription market will be over $155 billion in 2015 and grow further to $260 billion in 2020. In both countries, generics will increase their share of all prescriptions, but economic and structural incentives for new drug invention and brand-name prescribing by physicians will keep the share of patented drug sales high compared to countries with more direct government control over the pharmaceutical market.

**Conclusions:**

Expanding private insurance contributes to spending on branded drugs, since insurers compete for market share rather than cost savings. Health system reforms presently being enacted in the United States and China align to historical institutional trajectories in each country, but leave unresolved a core tension between incentives for new drug invention and universal access to affordable medicines.

## Introduction

Healthcare systems worldwide have been operating in a state of seemingly constant reform in recent years. The Unites States and China are expanding public and private insurance systems in an effort to broaden access to care and support preventive medicine. By contrast, most other developed and developing countries are advancing policies to stabilize or even reduce national health spending, especially on prescription drugs [1]. While the United States and China are at significantly different economic development levels, they share key features that make a comparison of healthcare reform initiatives and analysis of future prescription drug markets timely and relevant. First, they are the world’s two largest national economies and occupy the peak attention of strategic planners at multinational pharmaceutical and other global healthcare firms. Second, both are encountering policy tensions between access to care and costs of treatment as they broaden health insurance coverage. Third, both countries are beginning to seek ways of managing rising prescription drug costs after long periods of largely industry-set pharmaceutical pricing.

Every country necessarily strikes a balance between financial rewards for pharmaceutical innovation and ensuring public access to care. The United States allows free-market pricing of insurance and medicine for the majority of working adults, coupled to public insurance for the elderly and poor. Healthcare spending has expanded to over 17 percent of GDP and supports a large number of research-intensive biopharmaceutical and medical device firms. Even with expanded insurance availability under the 2010 Affordable Care Act, the system is fundamentally oriented to leading edge treatment, rather than the mass-scale delivery of inexpensive care [2]. Similarly, China in recent years has encouraged investments by leading pharmaceutical and medical device companies, both foreign and domestic. While a complex system of price controls keep doctor visits and hospital-based care affordable, the costs of many branded medicines and most diagnostic tests are borne by patients [3]. Both countries thus face policy tensions between wanting to incentivize domestic pharmaceutical research and avoiding unconstrained growth in healthcare expenditures. Yet, within these broad similarities, the two countries are following unique strategies in an effort to limit pharmaceutical price growth. In the United States, the government-run Medicaid program negotiates prices that require significant manufacturer discounting. Furthermore, the 2010 Patient Protection and Affordable Care Act (PPACA) includes measures to fund cost-benefit analyses of pharmaceuticals and promote greater generic drug use. In China, the central government has sequentially expanded an essential drugs list (EDL) that requires manufacturers to sell key medicines at prices intended to make them widely affordable.

This article describes the history of health system reform in the United States and China, with a focus on insurance coverage and incentives for pharmaceutical industry research and development (R&D). Policies to expand insurance are analyzed in light of each country’s institutional trajectory and projections are developed for national pharmaceutical markets through 2020. “Bottom-up” estimates are derived from spending on prescription drugs per patient, adjusted for changing population demographics, insurance coverage, and anticipated substitution of generics for branded drugs. “Top-down” figures are calculated from long-term growth trends in healthcare expenditure, adjusted for shifts in spending among hospitals (inpatient), specialty and private clinics (outpatient care), pharmaceuticals, and other forms of treatment. Results from the two methods turned out to be closely aligned for each country, providing additional confidence in the projections. Sensitivity analyses were carried out to highlight assumptions that underpin the findings and to clarify how policy implementation will influence each country’s pharmaceutical market. In turn, the estimates provide a baseline for analyzing the sustainability of drug price policies and evaluating future competitive dynamics in the pharmaceutical sector, especially between branded and generic drug producers.

Research findings and market projections developed here contribute to pharmaceutical policy studies and the analysis of health policy in several ways. First, the article describes contemporary reform initiatives through a historical perspective that explains the expansion of insurance coverage and other system changes underway in the United States and China. Second, we develop estimates for each country’s future pharmaceutical market based on trends in prescription drug use among subpopulations and as a percentage of total healthcare spending. Third, the discussion analyzes competitive dynamics within the pharmaceutical sector. By comparing and contrasting developments in the United States and China, the article offers unique insights on the two largest future markets for prescription drugs.

## Methods

This article presents a qualitative (describing the historical and institutional development) and quantitative (modeling future drug markets based on trend data and justified assumptions of growth rates) study of healthcare reforms and pharmaceutical markets in the United States and China [4]. The analysis draws on diverse sources: secondary literature in health policy and health economics; primary sources including government and commercial databases; interviews with executives at insurance, pharmaceutical, and medical device and diagnostics companies; and direct observations by the authors of healthcare in both countries.

The qualitative component of this study describes the historical development and path dependency of health insurance and pharmaceutical pricing policy for each country [5]. In the United States, public and private insurance have long operated independently and a large uninsured population has relied on emergency care rather than routine and preventive measures. The PPACA continues America’s unique mix of public and private insurance and private delivery of care without comprehensive price control mechanisms, even as it broadens coverage. China has an institutional history of patients paying out of pocket for most care, but at prices set by the government for doctor visits and most interventions. Reforms over the past decade have significantly expanded public health insurance and allowed private insurers to offer plans, but out of pocket payments remain high, especially for pharmaceuticals.

Building on the institutional trajectory analysis, the article develops estimates for each country’s future pharmaceutical market. The models are built on population demographics in each country and prescription drug use by age, insurance status, and treatment site. A second component to the models draws on the compound annual growth rate (CAGR) of healthcare expenditures in relation to the gross domestic product (GDP) and growth over time of prescription drugs within total health spending. To enable cross-national comparisons, we focus throughout on allopathic prescription drugs and exclude over-the-counter drugs, consumer health products, traditional Chinese medicines (TCM), and homeopathic or other alternative therapies from the analysis. In China, TCM hospitals and clinics often prescribe both allopathic and TCM drugs; they are therefore included as a treatment location for projections of pharmaceutical sales. Although the figures are necessarily estimates, calculating market size in two distinct ways provides an internal confidence test. Sensitivity analysis for each of the four projections (micro and macro for the United States and China) draws attention to key policy parameters that will shape future pharmaceutical markets.

The discussion draws on the study’s qualitative and quantitative components to analyze future competitive dynamics, especially between branded and generic drug companies. The article enables comparison of the world’s two largest pharmaceutical markets, revealing similarities in institutional trajectories despite major differences between the health systems and overall economic development of the United States and China.

## Insurance, pricing, and biomedicine in the USA

In contrast to primarily government-run or social insurance models found in most OECD countries, the United States has a history of private group health insurance, originating with plans offered by hospitals and Blue Cross and Blue Shield starting in the early 1930s [6]. Since that time, health insurance companies specialized distinct from property and life insurance. By 1951, over half of the patients admitted to hospitals held private medical insurance [7]. American physicians long opposed insurance coverage for visits to private practices, fearing interference in their professional domain. Yet insurers gradually began covering outpatient visits and prescription drugs starting in the 1960s. As the group coverage model expanded, insurers focused on effective pricing for employers and restricted individual plan offerings to healthier and wealthier participants. Insurers competed to attract members least likely to run up costs and managed spending by declining coverage for pre-existing conditions and setting coverage limits. In order to attract companies that selected and subsidized insurance choices for their employees, insurers expanded portfolios of covered care, including ever-broader prescription drug formularies.

Public insurance was proposed in the United States at numerous historical moments of welfare system expansion, but failed to gain Congressional approval through the first two-thirds of the 20th century. The creation of Medicare and Medicaid in 1965 and subsequent expansion of coverage provided public insurance for the elderly, disabled, and working poor [7]. By accepting all elderly and poor Americans, these programs unintentionally further supported the dynamic of private insurers competing on the basis of group size and numbers of employers covered, rather than by controlling costs or managing disease for pre-retirement individuals. While the original Medicare program did not cover prescription drugs, reforms in 2003 expanded government insurance for pharmaceuticals, albeit with significant co-payments [8]. The outward complementarity of private and public insurance nevertheless left major coverage gaps. Some were by choice, notably among younger Americans who elected not to buy insurance. But in many instances self-employed or part-time workers found private individual insurance plans unaffordable even as they failed to qualify for public insurance.

Although coverage gaps and above inflationary cost increases have been a perennial topic of concern, health spending in the United States correlates to a world-leading position in biomedical research and pharmaceutical and biotechnology industries. Spending on R&D in the United States by pharmaceutical firms exceeded $48 billion in 2012, and they directly employed over 700,000 Americans while providing 2.5 million jobs in supporting industries [9]. Eight of the top fifteen global pharmaceutical and biotechnology firms are headquartered in the United States, and all of the top firms have research labs in the country. For smaller biotechnology firms, the United States has an even more dominant lead, with some 1,500 firms employing nearly 200,000 people. Overall, 14 percent of the U.S. working population is employed by healthcare industries, including in insurance and the provision of care.

The PPACA was enacted in 2010 against this institutional backdrop and generated vigorous debates regarding the distribution of healthcare spending, benefits and risks of broadening access to care, and the role of government in insurance. As approved, the PPACA provides for expanded access to government-backed insurance under Medicare and Medicaid and mandates the purchase of private insurance by individuals not covered through employers or other programs [10]. Its stated goal is to provide insurance to the 45 million Americans that were uninsured in 2010. The PPACA thus signals an important break from a history that left between 10 and 15 percent of the population uninsured at any given time. Yet, it left unchanged the core institutional trajectory of parallel but uncoordinated public and private insurance systems. Some provisions tackle disproportionate health spending in the United States, notably the creation of accountable care organizations with incentives for physician groups and hospitals to deliver care at below the prevailing average cost [11]. At the same time, the PPACA’s expansion of insurance will increase health spending in other areas, notably on pharmaceuticals and medical devices and diagnostics.

### Projections for the U.S. pharmaceutical market

Three major provisions of the PPACA are key to analyzing its likely effect on the composition of future healthcare spending. First, young adults up to age 26 can remain on their parents’ group insurance. Second, Medicaid and Medicare will expand the number of people insured and the amount of care that is covered. Third, insurance marketplaces (termed “exchanges” in the Act) are being established for citizens and legal residents between age 26 and 64 to purchase private insurance. Government subsidies offset some insurance costs, scaled to income levels. Full implementation the PPACA will unfold over the course of several years and the number of people ultimately gaining coverage will depend on state-level policy decisions and purchasing choices by individuals. Since penalties for failing to obtain coverage are modest for 2014 and 2015 but rise to 2.5 percent of income by 2016, new insurance enrollment will spread out over several years. To estimate how the PPACA will affect spending on pharmaceuticals specifically, this article draws on estimates of demographic shifts aligned to public versus private insurance, changes to drug prices and sales volumes, and prescription drug purchases by the insured in different insurance pools.

#### Consumption-based estimates

Pharmaceutical use at an aggregated national level relies on patients’ age and insurance status. Unsurprisingly, older Americans on average consume far more prescriptions than younger age groups. Likewise, people with insurance take more medicine than the uninsured. The insured also are written greater numbers of prescriptions for more expensive branded drugs thanks to doctor awareness of insurance status and because patients request particular medicines [12]. Consequently, demographic shifts associated with the retirement of the baby boom generation and the PPACA’s insurance coverage mandates will combine to expand total drug sales. Calculating the future pharmaceutical market based on the drug-consuming population therefore relies on three major variables: the population age profile, drug spending by insurance status, and drug price and sales volume changes over time. See Table 1 for pharmaceutical spending by insurance status and age cohort in the United States with our projections through 2020.

**Table 1:** **Prescription drug expenditures by insurance and age in the United States**

	2010		2015		2020	
	Population	Rx (per capita)	Population	Rx (per capita)	Population	Rx (per capita)
Under 26	108,528,000		110,205,000		110,680,000	
Private insurance	63,602,000	209	68,737,000	300	69,033,000	431
Public insurance only	32,192,000	368	37,139,000	528	37,631,000	758
Uninsured	12,734,000	109	4,329,000	156	4,016,000	225
26-64	158,889,000		163,463,000		167,247,000	
Private Insurance	115,778,000	915	127,838,000	1,314	136,923,000	1,886
Public Insurance only	15,619,000	2,376	22,558,000	3,411	23,582,000	4,897
Uninsured	27,492,000	275	13,068,000	395	6,742,000	567
65 and over	41,158,000		47,695,000		55,969,000	
Medicare only	15,683,000	2,036	18,174,000	2,923	21,327,000	4,196
Medicare and private	20,799,000	2,350	24,102,000	3,374	28,284,000	4,843
Medicare and other public	4,062,000	2,953	4,707,000	4,239	5,524,000	6,086
Uninsured	614,000	1,577	711,000	2,264	835,000	3,250
Total	308,575,000	270,907	321,363,000	446,956	333,896,000	699,517

*Note:* Population is actual or projected number rounded to nearest thousand; total prescription drug (Rx) spending is in millions of US$, nominal.

*Sources:* U.S. Census Bureau, “Projections of the Population by Selected Age Groups: 2010 to 2050”, (May 2013); Agency for Healthcare Research and Quality, “Medical Expenditure Panel Survey: Prescription Medicines Expenses per Person by Source of Payment” (May 2013).

Beginning with the first variable, shifting age demographics will have a major impact on the future drug market. The PPACA has clear age cohorts for insurance coverage mandates. Since 2011, young adults up to age 26 can remain covered under their parents’ private insurance plans. Starting in 2014, young adults that historically opted out of insurance at high rates will be penalized for failing to obtain coverage. Demographically, this population is growing slowly in the United States and has low total drug spending. Once an initial wave of new coverage for young adults levels off, drug spending among this population will grow modestly. For even younger Americans, the Children’s Health Insurance Program (CHIP) operated by Medicaid will expand under the PPACA. Based on Congressional Budget Office estimates, we estimate that Medicaid and CHIP will add an estimated 4.4 million under age 26 to public insurance by 2015 and nearly 4.8 million by 2020 [13]. Polls of those opposing implementation of the PPACA suggest that significant numbers of the uninsured between 18 and 26 will continue to opt out of insurance purchases. Our estimates thus add only 50 percent of the present uninsured to private insurance, but retain others as uninsured.

The highest variability for insurance coverage is in the second cohort, aged 26 to 64, who are required under the PPACA to obtain coverage through an employer-offered plan, Medicaid, or through new state-based insurance exchanges. While the PPACA mandates universal coverage, of the present 32 million uninsured in this cohort, many will remain uninsured through 2020. Large numbers of uninsured are a product of policy decisions by nearly half of the states not to establish insurance marketplaces, though people can purchase private insurance through other channels. In addition, 25 states declined the initial round of federal funds for Medicaid expansion. Our projections therefore add 9 million people net to private insurance by 2015 and 15 million by 2020. Under these estimates, Medicaid coverage will expand by 6.5 million by 2015 and more slowly thereafter with 7.2 million new enrollees by 2020. These figures comport with evidence that a total of 8 million Americans enrolled on health exchanges by April 2014, the majority of whom were not previously insured [13].

All Americans 65 and over are eligible for Medicare, and the PPACA broadens prescription drug coverage to eliminate a coverage gap in the 2003 Medicare Part D legislation (under which seniors were responsible for annual drug costs above $2,830 but below $4,550). As the baby boom generation joins Medicare, the number of Americans in this category will grow quickly. To be conservative, estimates here keep the ratios constant between seniors with only Medicare and those who purchase supplemental coverage. As out-of-pocket spending declines for seniors thanks to the expansion of Medicare Part D, branded drug sales will rise and overall drug spending for seniors may grow even beyond these projections.

The second variable, prescription drug spending, varies by insurance status with clear distinctions among the uninsured, privately insured, and publicly insured. According to U.S. government statistics, the average prescription drug spending per person under age 65 in 2010 was $223 for the uninsured (paid out-of-pocket or by various public sources and charities), compared to $664 for the privately insured and $1,024 for those covered by Medicaid or other public insurance. The subgroup weighted average prescription drug spending per person in 2010 was $244 for those younger than 26 and $948 for adults between 26 and 64. Retirees covered by Medicare spent between $2,036 and $2,953; those at the higher expenditure level held supplemental insurance^a^. The population-weighted average of pharmaceutical spending among retirees was $2,278. Overall, as the number of uninsured working adults drops and Medicare Part D eliminates out-of-pocket copayments, average per capita spending will grow significantly.

The third variable is the rising price and sales volume of prescription drugs. Pharmaceutical sales historically have grown faster than inflation, though generic substitution slowed the total market expansion from nearly 11 percent annually in the 1990s to 8 percent each year between 2000 and 2010, to 3 percent in 2011, and to a 1 percent decrease in 2012^b^. In addition to the shift to generics underway, the recession that started in late 2007 led to a decline in the rate of growth of total U.S. health spending [14]. A resurgence of new drug approvals in 2012 and fewer blockbuster drug patent expirations in the near term will combine to drive drug prices to pre-recession growth levels. At the same time, the volume of prescriptions will rise considerably for the newly insured. To account for both volume and price growth, we project spending per capita on drugs to grow at 7.5 percent yearly for each of the age groups.

#### Macroeconomic trend-based estimates

A second method for estimating the future U.S. pharmaceutical market is derived from macroeconomic trends in total healthcare spending and the division of spending among different forms of care. Our estimates build on data from the Centers for Medicare and Medicaid Services (CMS) by adding macroeconomic projections of healthcare expenditure as a percentage of GDP, and sector-level projections based on the U.S. pharmaceutical market’s growth over time.

Between 1980 and 2010, health spending in the United States grew by a CAGR of 8 percent; as a percentage of the GDP, healthcare expanded from just over 9 percent to nearly 18 percent. Table 2 shows macroeconomic figures for GDP and health spending in the United States since 1980 and our projections through 2020. Implementation of the PPACA will drive greater total national health spending thanks to broader insurance coverage. But growth will be uneven across the major categories of spending. Inpatient hospital spending will level as emergency room expenditures decline as a result of the presently uninsured gaining access to routine primary care. Other savings will be realized when insurers negotiate lower fees for inpatient and outpatient medical services than are billed to uninsured individuals. The rapid expansion of “narrow networks” in 2014 supports these forecasts.

**Table 2:** **U.S. GDP and major categories of healthcare expenditure (US$, millions, nominal)**

	U.S. GDP	Total health spending	Prescription drugs	Hospital care	MD & outpatient
1980	2,788,000	255,700	12,000	100,500	47,700
1990	5,801,000	724,000	40,300	250,400	158,900
2000	9,951,500	1,378,000	120,900	415,500	290,000
2010	14,498,900	2,593,600	270,900	815,900	519,100
Projected 2015	17,722,000	3,544,400	442,050	1,134,200	673,000
Projected 2020	22,145,000	5,204,100	702,550	1,665,300	936,700

*Sources:* Economist Intelligence Unit, “GDP: United States of America”, http://www.eiu.com, May, 2013; U.S. Centers for Medicare and Medicaid Services, “National Health Expenditure Data”, http://www.cms.gov, May 2013.

Using very optimistic growth projections for the overall economy, CMS has forecast total healthcare spending to rise to 18.3 percent of GDP in 2015 and to remain slightly below 20 percent of GDP in 2020 [15]. However, the economic recovery slowed in mid-2011, and recent evidence suggests the GDP continues to grow at a modest rate. At the same time, the historically unprecedented slowdown in healthcare expenditures witnessed during the initial part of the recent economic recession will reverse as the large baby boom generation accesses Medicare and previously uninsured individuals make greater use of primary care. The first quarter of 2014 thus featured a 9.9 percent rise in total healthcare spending.

By 2015, we estimate approximately 35 million Americans will be newly insured by Medicare, Medicaid, through their employer, or by purchasing coverage under the PPACA. By 2020, an additional 14 million Americans will join Medicare and an additional 10 million will have at least partial coverage under Medicaid [16]. This coverage expansion will drive overall health spending at historical rates, even with cost efficiencies from more efficient care. Total healthcare spending will be over $3.5 trillion in 2015, making up 20 percent of GDP. Spending will rise further to $5 trillion in 2020, resulting in healthcare contributing 22.5 percent of the U.S. GDP.

The prescription drug market grew by a CAGR of nearly 11 percent from 1980 to 2010, although it slowed to 8 percent annually over the decade that started in 2000. Within overall healthcare expenditure, drugs have grown by 2 to 3 percent annually on average since 1980. As a consequence, spending on hospitals and primary care physicians declined slightly as a percentage of total healthcare expenditure over the past two decades. The same combination of national demographics and changes to coverage under the PPACA that will drive greater total healthcare spending also will lead to greater spending on pharmaceuticals relative to other forms of care. Economic incentives and policy changes will drive the newly insured and elderly with greater prescription drug coverage under Medicare to consume more prescriptions at the expense of outpatient care spending. Thus the macro-based trends strongly suggest that recent declines in pharmaceutical spending will reverse and the prescription market will return to above-inflationary growth.

## Insurance, pricing, and biomedicine in China

Throughout the 19th and early 20th centuries, Chinese patients typically paid providers directly for medical care. Following the 1949 establishment of the People’s Republic of China, medicine was provided as a public good. Private firms and market incentives were eliminated, resulting in collective (government) ownership and management of hospitals, drug manufacturers, and other components of care. Beginning in 1978, a gradual but sequential set of reforms encouraged the formation of township and village enterprises, created special economic zones for export-oriented businesses, and partially liberalized healthcare [17]. Pharmaceutical prices were freed in the 1980s. As prices rose, out-of-pocket spending increased from 21 percent to 60 percent of all health expenditures by 2000. The government’s share, by contrast, shrank from over 36 percent to 14 percent. Nevertheless, hospitals remained under state ownership and few private clinics were opened.

Starting in the mid-2000s, the Chinese government initiated significant policy developments in the medical system. National, provincial, and municipal funds were used to underwrite new insurance coverage and to build additional medical infrastructure of hospitals and research facilities. Three public insurance plans were developed incrementally: the New Cooperative Medical Scheme (NCMS) for rural residents, the Urban Employee Basic Medical Insurance (UEBMI) for working adults in cities, and the Urban Residents Basic Medical Insurance (URBMI) for children, retirees, and others in cities. All three programs feature low annual premiums (approximately $20 for NCMS and modestly higher for urban residents). Coverage includes inpatient expenses, basic physician consultations, and medicines on the EDL. Most other health spending in China remains out-of-pocket, with the potential for overwhelming costs if patients with basic insurance are diagnosed with cancer or other complicated diseases [18, 19].

By 2013, basic insurance programs covered over 95 percent of the population. Seeking to balance the economy from high savings and investment to broader consumption, the government announced new funds for hospitals, specialty clinics, and basic biomedical research. Oversight for the health system was centralized in the new National Health and Family Planning Commission (H&FPC), which incorporated the former Ministry of Health and the National Population and Family Planning Commission.

Even as the government introduced and broadened the availability of health insurance, a longstanding system of price controls on doctor visits, surgery, and other procedures ensured wide access to basic care. In turn, the low cost of care is made possible through markups on diagnostic tests and pharmaceuticals, which patients pay out of pocket. Over the past two decades, pharmaceutical sales grew to account for 40 to 50 percent of hospital revenue and physicians came to earn some 30 percent of their pay through profit sharing with hospitals. A series of policy interventions since 2007 sought to cut pharmaceutical prices for consumers, either through government subsidies for medicines declared “essential”, or regionally fixed prices for treatments for common infectious diseases and chronic care medicines for diabetes, heart disease, and certain other conditions [20]. Incentives thus aligned for extensive prescription of branded drugs and widespread use and over-use of diagnostic tests [21]. Alongside supplier-induced demand, imported pharmaceuticals and diagnostic tests also enjoy market pull from a growing middle class and wealthy urban population.

Historically, provincial administrations in China promoted local firms, leading to a patchwork of over 5,000 small-scale generic drug producers, most of which were state owned. Industry concentration remains low, with the top 100 firms only accounting for one-third of national drug sales. While some consolidation has taken place in the past five years, new domestic research-oriented companies also are being founded at a rapid rate, often with project collaborations and advisory board members from the United States and Europe.

The recent growth and future prospects of China’s healthcare market also have attracted investment from a more concentrated multinational pharmaceutical industry. Over half of the top 20 global pharmaceutical firms have built R&D facilities in China, and together with numerous smaller firms are hiring China’s world-leading numbers of new Ph.D. chemists and biomedical scientists [22]. The role of venture capital has also grown in China, with 70 VC-backed biotechnology companies present in 2012, more than double that of any other emerging market [23].

### Projections for the Chinese pharmaceutical market

Total health spending in China was modest through recent history, especially when compared to the United States or other developed countries. Spending on all care averaged only $21 per person in 1995, but then grew at a CAGR of 17 percent to $190 in 2009. Reforms that broadened insurance and increased health infrastructure spending combined to accelerate spending growth by 35 percent a year to $350 per person in 2011 [24]. Spending on prescription drugs in China also began to rise in the mid-1990s, and has expanded subsequently at an average annual rate of 20 percent. Total pharmaceutical sales were RMB 580 billion ($92 billion) in 2012 and an estimated RMB 695 billion ($112 billion) in 2013, ranking China’s prescription drug market second worldwide after the United States^c^. Over the same period, concerns about the quality of domestic medicines and financial incentives for hospitals and doctors to prescribe expensive drugs contributed to a shift to branded pharmaceuticals manufactured by multinational firms and joint ventures. Generic drugs have continued to grow in sales volume, but have experienced a market share decline from 80 percent of all drug sales in 2005 to 60 percent in 2010.

To estimate the effects of ongoing reforms and the expansion of insurance coverage for pharmaceutical spending, we developed projections based on prescription sales at the location of care and as a percentage of total healthcare expenditure. Prescription drug spending will grow rapidly thanks to continued urbanization, an ageing population profile, and rising wealth levels. At the same time, pharmaceuticals will make up a smaller percentage of total healthcare expenditure, especially as fees for hospital and doctor services are partially liberalized under policy changes announced in 2013. On average, prices for drugs will likely drop, especially as the EDL is implemented nationally. The EDL, which grew from 300 compounds in 2009 to 500 in 2013, now includes both generic and branded drugs for which manufacturers gain greater national sales but at significantly lower prices [25]. Over 100 of the medicines on the present EDL are sold by multinational pharmaceutical firms with growing R&D investments in China; the rest are generics manufactured and sold by one or more domestic producers.

#### Consumption-based estimates

Because health insurance is a new development in China, no historical data exist for drug spending by insurance status. To develop “bottom-up” projections for the future pharmaceutical market in China we instead analyzed drug sales by location of patient care, including general hospitals, specialized hospitals, community health clinics (CHCs), township health clinics (THCs), and TCM hospitals and clinics. Two key variables are used to calculate the future pharmaceutical market: numbers of individuals treated in various hospitals and clinics, and the average drug expenditure per patient visit. Table 3 shows visits to hospitals and other clinics in China, with projections through 2020.

**Table 3:** **Hospital and medical clinic patient visits in China (thousands per year)**

	General hospitals		Specialized hospitals		CHCs		THCs		TCM	
	Inpatient	Outpatient	Inpatient	Outpatient	Inpatient	Outpatient	Inpatient	Outpatient	Inpatient	Outpatient
2008	58,720	1,282,280	5,500	132,500	1,410	255,590	33,550	828,450	8,890	266,110
2009	67,130	1,368,482	6,020	164,448	2,250	374,725	38,080	838,528	10,340	291,118
2010	75,050	1,435,532	6,550	175,897	2,620	481,896	36,300	837,901	11,680	316,022
2011	84,310	1,589,690	8,440	179,560	2,470	409,000	34,490	832,010	13,490	347,510
2012	93,584	1,700,968	9,706	201,107	2,978	478,391	33,628	811,210	15,502	379,842
2015	127,989	2,083,759	14,762	282,541	5,216	765,531	31,168	751,877	23,523	496,031
2020	196,926	2,723,390	26,601	455,035	13,252	1,607,876	24,117	581,788	47,108	773,901

*Source:* People’s Republic of China (2012) *Health Statistical Yearbook*, Beijing: P.R. China.

Ranked by the government on a scale from tier-III (larger and better-equipped) to tier-I (smaller and less-well resourced), general hospitals provide both inpatient and outpatient care [26]. Hospital pharmacies are the primary site for pharmaceutical sales in China. Widespread basic insurance, urbanization, and access to information about hospitals and clinics in on-line forums have combined to significantly increase the number of patients seeking care at general hospitals. In turn, greater inpatient and outpatient care at general hospitals has resulted in more prescriptions of branded pharmaceuticals. New hospital construction is proceeding fast in larger urban settings. However, rapid urbanization also has led to overcrowding of existing hospitals, while access to newly built hospitals is limited by their locations at the outer edge of cities and fee structures. These factors suggest that the growth of inpatient visits to general hospitals will slow from the present 13 percent expansion annually to 11 percent through 2015 to 9 percent thereafter. Outpatient care at general hospitals also will experience slightly lower rates of growth. At the same time, new specialty clinics will attract rising numbers of patients, especially people with greater financial resources or with private insurance to cover more expensive care. For inpatient care at specialty clinics we therefore project a CAGR of 15 percent through 2015, which will subsequently moderate to 12.5 percent annually. Outpatient care at specialty clinics will rise through 2015 at 12 percent annually and at a slightly lower rate of 10 percent thereafter.

Community health clinics (CHCs) have long served as intermediaries between even smaller clinics and full service general hospitals. As China urbanized in recent decades, however, many CHCs fell into disrepair and patients instead sought out care in large hospitals. Seeking to manage patient volumes at general hospitals, provincial and municipal governments recently began to invest in CHCs to turn them into primary care sites. Under this model, patients with serious illnesses would be transferred to tier-II or tier-III hospitals while the CHC system would provide faster primary care and reduce the present strain on general hospitals. Government officials also view the growth of CHCs as a way to manage spending. Physicians working at CHCs are paid less than at general hospitals, and CHCs appear more willing to purchase domestically sourced diagnostic instruments and devices. Inpatient numbers at CHCs and other primary care facilities are therefore projected at a CAGR of 20.5 percent in coming years, while outpatient volumes will continue to grow at the present rate of 17 percent through 2015 and slightly slower at 16 percent thereafter.

Even as top-tier urban hospitals and CHCs experience rapid growth, township and rural clinics are experiencing declining patient numbers. While township health clinics (THCs) will continue to provide basic care in smaller towns and rural settings, patients with insurance typically seek out care from CHCs and general hospitals. Patient numbers will decline at an accelerating pace, from 2.5 percent fewer inpatient and outpatient visits through 2015 to a 5 percent decline annually thereafter. Furthermore, while inflation will continue to push up average expenses for care, we project that greater use of domestically manufactured generic drugs in THCs will bring down pharmaceutical spending per patient in coming years.

TCM typically is delivered by dedicated departments within general hospitals or by physicians in private practice and small group clinics. Thanks to government support for TCM, greater attention to individual patients at TCM clinics, and pride in China’s medical heritage, TCM presently is experiencing growth. Furthermore, government policies support the co-prescribing of allopathic and TCM medicines at general hospitals and TCM specialty clinics. Present efforts to standardize TCM drugs, develop new TCM medicines – including in collaboration with multinational pharmaceutical firms – and demonstrate efficacy through clinical trials are likely to increase TCM sales significantly in coming years and will further drive growth of the prescription drug market. Nonetheless, the focus of new hospital and clinic construction is on allopathic and integrated sites, not TCM-specific hospitals. Thus, care at TCM-only facilities will generate only modest additional pharmaceutical sales in coming years.

Regulatory interventions announced in 2012 and 2013 will change the growth rate of drug prices and numbers of prescriptions written. First, policies that allowed hospitals a 15 percent markup on prescription sales are being amended [27]. Some provinces are banning excessive pharmaceutical profits and the H&FPC has significantly expanded the EDL, on which only a modest additional pharmacy fee is permitted. Second, prospective payment systems and diagnostic related groups are being developed by a coalition of insurers and the government. The government is likely to implement more broadly programs that bundle payments for care. Pioneered in several cities, bundled payments reduce pharmaceutical expenses while costs for physician consultations and surgery rise [28]. Third, the government is developing policies to increase physician compensation and loosen price controls on hospital services. Hospitals and clinics will become less dependent on branded drug sales to offset other expenses. The consequences of these policy developments for spending on medicines by the location of sales are presented in Table 4, with projections through 2020.

**Table 4:** **Prescription drug expenditures in china by treatment site (US$, billions, nominal)**

	General hospitals		Specialized hospitals		CHCs		THCs		TCM		Total Rx
	Inpatient	Outpatient	Inpatient	Outpatient	Inpatient	Outpatient	Inpatient	Outpatient	Inpatient	Outpatient	
2011	38.2	22.8	4.6	2.8	0.4	3.5	2.6	3.3	0.6	1.0	79.9
2012	46.8	26.8	5.9	3.5	0.5	4.2	2.6	3.3	0.8	1.2	95.4
2015	81.8	41.2	11.4	6.1	0.8	6.6	2.4	2.9	1.4	2.1	156.8
2020	148.7	61.9	24.3	11.4	1.5	10.8	1.5	1.7	3.5	4.4	269.6

*Notes:* Figures initially calculated in RMB (Renminbi), converted to US$ using actual rates through 2013 and projections of $1: RMB 5.8 for 2015 and 1$: 5.5 RMB for 2020. These align to market expectations for exchange rates and policy statements from the People’s Bank of China. Total prescription drug sales for 2011 and 2012 differ from Table 5 due to discrepancies in the original data sources.

*Source:* People’s Republic of China (2012) *Health Statistical Yearbook*, Beijing: P.R. China.

As a result, our model forecasts drug spending as a percentage of the total care expense per patient visit to fall by 2 percent annually for general and specialty hospitals through 2015 and by 5 percent CAGR through 2020. At CHCs, pharmaceutical spending as a ratio of the total bill for care will fall by 3 percent annually through 2015 and by 6 percent CAGR through 2020. Even as THCs experience fewer patient visits, they too will see drug spending fall as a ratio of the healthcare bill by 4 percent annually through 2015 and by 6 CAGR through 2020. TCM care, by contrast, will see pharmaceutical spending level through 2015 but decline relative to other expenses by 2 percent annually through 2020, even as the number of patients treated and prescriptions written rises.

#### Macroeconomic trend-based estimates

A second method for estimating China’s future pharmaceutical market is derived from macroeconomic trends in total spending on health and its allocation among care segments. China’s nominal GDP has grown at a remarkable rate in recent decades, at a CAGR of 14.3 percent between 1990 and 2010. Growth slowed to 7.8 percent in 2012 and fell further to 7.5 percent in 2013 [29]. To be conservative, we project total economic growth at 7.5 percent annually through 2020. This estimate is backed by government estimates and reflects slower growth as China shifts from an export and investment-based economy to greater domestic consumption.

Healthcare expenditure as a percent of GDP has expanded even as China recorded double-digit economic growth. In 2000, health spending accounted for 4.6 percent of GDP. It then rose steadily to 5.1 percent in 2012. Table 5 presents macroeconomic data for China's GDP and health spending since 2000 and our projections through 2020. In nominal terms, spending on health increased from $55 billion to $420 billion. As disposable income grows, the population ages, and the country shifts further to a consumption-oriented economy, total healthcare expenditure will increase significantly. At the same time, policymakers are concerned with inequalities that could arise from overly rapid liberalization of healthcare prices. We therefore project total healthcare expenditure to account for 7 percent of GDP in 2015. Growth will moderate thereafter such that healthcare contributes 8.5 percent to the GDP in 2020. At that rate, total spending on health will match projections by government officials and external analysts of RMB 8 trillion ($1.3 trillion) in 2020 [30].

**Table 5:** **China’s GDP and pharmaceutical expenditure (US$, millions, nominal)**

	China GDP	Total health spending	Healthcare as % of GDP	Prescription drugs
2000	1,198,475	55,404	4.6%	13,850
2005	2,257,619	105,716	4.7%	25,340
2010	5,931,203	295,153	5.0%	60,270
2011	7,324,952	369,910	5.1%	77,300
2012	8,226,885	419,571	5.2%	91,980
2015	10,220,234	715,416	7.0%	158,940
2020	14,672,467	1,247,160	8.5%	261,840

Sources: People’s Republic of China (2012) *Health Statistical Yearbook*, Beijing: P.R. China; People’s Republic of China (2012) *China Statistical Yearbook*. Beijing: P.R. China.

Within total health spending, pharmaceuticals grew at a world-leading rate throughout the 2000s, averaging 19 percent CAGR growth over the decade. In 2011 and 2012, pharmaceutical sales jumped by 20 percent. While appealing to multinational pharmaceutical firms, who have responded by investing in research centers, increasing clinical trials in China, and undertaking joint ventures with Chinese firms, pharmaceutical sales growth is likely to slow in coming years for two key reasons.

First, the restructured H&FPC is expanding the EDL for therapies that will be produced by domestic generic drug companies and available nationally [25]. The EDL effectively puts a price cap on pharmaceuticals, slowing annual drug spending increases. In the early 2000s, price caps in some provinces led to undersupply and shortages of medicines [31]. However, given the considerable attraction of the Chinese market to pharmaceutical firms, most appear willing to produce medicines on the EDL at very low profit in order to benefit from access to the market for other branded drugs. Second, attention to unethical behavior in the distribution of pharmaceuticals and accusations of bribery to secure contracts with hospitals is prompting intensified regulatory scrutiny. In early 2014, several domestic and multinational firms were charged for bribing hospitals and doctors. New measures are being implemented by national and provincial authorities to make pharmaceutical purchasing more transparent and competitive.

Overall, the volume of pharmaceutical use will continue to expand, especially as the average cost of a prescription falls. China’s population growth rate has been a modest 0.6 percent over the past decade and the number of people over age 60 rose from 8.6 percent of the population in 1990 to 12.4 percent in 2010. By 2020, 17 percent of the population will be over 60 [32]. As in other countries, elderly Chinese will be prescribed more medicines and will be drivers of greater total spending on pharmaceuticals. Nevertheless, concerted government efforts to balance healthcare spending will lower the annual pharmaceutical growth rate to 10.5 percent by 2020.

## Results

In the United States, the “bottom-up” forecasting method that draws on prescription drug use by insurance status predicts a total pharmaceutical market of $447 billion in 2015 and $699.5 billion in 2020. Using the macroeconomic approach, the U.S. pharmaceutical market is projected to rise to $442 billion in 2015 (comprising 12.5 percent of all health spending) and to $702.5 billion in 2020 (making up 13.5 percent of all healthcare spending). For 2015, the two methods vary by 1 percent; for 2020, they differ by 0.4 percent.

In China, since the total patient numbers will continue to rise quickly across the major care facilities, overall spending on prescription drugs will continue to grow significantly. Calculated by the site of care, pharmaceuticals will grow to RMB 910 billion ($156.8 billion) in 2015 and RMB 1.5 trillion ($269.6 billion) in 2020. As a result of a shift from very high annual increases in prescription drug spending to greater growth in outpatient and inpatient care, total drug expenditure when calculated from macro trends will be $158.9 billion in 2015 and $261.8 billion in 2020. For 2015, the two methods vary by 1.3 percent; for 2020, they differ by 3 percent.

A sensitivity test on the U.S. microeconomic model reveals that variance in the uptake of insurance will result in relatively modest changes to the total pharmaceutical market. At an extreme, if the PPACA were repealed and the rate of uninsured working-age Americans held constant at 2010 levels, then prescription drug sales would be 6.7 percent lower than projected in 2015 ($417 billion) and 7.8 percent lower by 2020 ($645 billion). Alternatively, if all presently uninsured Americans obtain coverage, the market would grow by an additional 3 percent relative to our projections in 2015 ($460 billion) and 1.5 percent by 2020 ($710 billion). The model is more sensitive to drug price and volume changes. If prices and use rise by 11 percent (as they did on a compounded annual average basis between 1980 and 2010), then the market would grow by 17 percent relative to the projections in 2015 ($525 billion) and by 38 percent by 2020 ($964 billion). Alternatively, if prices and prescription drug use grow by a far more moderate 5 percent, the market would shrink relative to our model to $397 billion in 2015 and $553 billion by 2020.

For sensitivity analysis of the U.S. macroeconomic model, we varied total economic growth, healthcare as a percentage of GDP, and pharmaceutical sales as a percentage of total health spending by 10 percent each. Higher values across the board generated 33 percent greater pharmaceutical spending relative to our estimates ($589.7 billion in 2015 and $935 billion in 2020), whereas the lower values resulted in 27 percent lower spending than we project ($323 billion in 2015 and $512 billion in 2020). Changing only the total healthcare spending variable or the percentage of healthcare spent on pharmaceuticals generated equivalent percentage changes to total prescription drug sales.

A sensitivity analysis of the consumption-based model for China draws attention to the importance of treatment location and projected changes in drug prices and use. We varied patient visits and prescription drug expenditure by plus or minus 10 percent for each treatment site. If patient visits and drug expenditure per patient both rise by 10 percent relative to our projections, the total market will be 21 percent larger than our forecast in 2015 ($190 billion) and 15 percent greater by 2020 ($309 billion). By contrast, if both shrink by 10 percent compared to our estimates, the total pharmaceutical market will be 19 percent smaller than projected in 2015 ($127 billion) and 23 percent less by 2020 ($207 billion). Within these groupings, inpatient care in both general hospitals and specialized hospitals has a higher degree of sensitivity compared to other categories. If the EDL results in 10 percent decreased drug spending but continued rising patient volumes, the total market will be 10 percent lower than projected in 2015 and nearly 15 percent less by 2020.

For the China macroeconomic model, we varied GDP growth rates, healthcare spending, and pharmaceutical sales as a percentage of health spending by 10 percent each. Higher growth across all three variables would result in a pharmaceutical market 21 percent larger than estimated ($192 billion in 2015 and $317 billion by 2020). Lower growth produces a pharmaceutical market 19 percent smaller than our estimates ($129 billion in 2015 and $212 billion by 2020). Interestingly, changes to the growth rate of health spending and the percentage of spending going toward pharmaceuticals had relatively larger impacts than changes to GDP growth estimates.

## Discussion

Health systems worldwide struggle to balance delivery of care to the sick, support for expensive new products and services, and the conflicts of interest and moral hazards associated with insurance. In most countries, spending on healthcare is now seen as a cost to GDP, not a contributor to national prosperity. Healthcare reform thus involves a difficult choice between affordable present-day care and incentives for firms that develop new medicines, diagnostic tests, and imaging devices. The U.S. healthcare system confronts contradictory mandates to broaden insurance availability, maintain ready access to primary care physicians, and sustain world-leading investments in biomedical research. China’s healthcare system faces contradictory mandates of increasing access to care for a population of 1.35 billion, improving the quality of services, regulating the safety and efficacy of medicines, and providing incentives to research-based pharmaceutical, device, and diagnostics firms. Intriguingly, China and the United States are implementing reforms that expand insurance coverage and increase the private sector’s role in healthcare insurance and delivery even as other countries seek to reduce spending through greater direct government ownership of insurance and even the delivery of care. At the same time, reforms in both the United States and China are introducing significant new regulations that suggest global convergence toward price controls, whether by explicit mandate or through regulation of cost – efficacy tradeoffs.

Expansion of the health system in each country is shaped by historical path dependency. The U.S. PPACA builds on a trajectory of free-market pricing and opposition to government mandates for generics use or cost assessments as part of drug approval decisions. In China, reforms are broadening access to basic insurance, plans are being developed to expand government-backed coverage for high-cost medical events, and private insurance companies are expanding offerings. These policies fit with the country’s incremental policy reform that has sought to use market incentives to promote investment and innovation without undermining social cohesion. Even as prescription drug markets grow in both countries, however, tensions concerning drug spending will shift the balance between generics and branded pharmaceuticals.

### Pharmaceutical market dynamics

In the United States, generic drugs expanded from 30 percent of all prescriptions dispensed in the 1980s to 75 percent in 2010 [33]. Further gains in market share seem assured. Patent expirations on top-selling (“blockbuster”) drugs that began in 2010 will continue through 2016, adding competitors to drugs averaging $21.7 billion in sales per year. In addition, the Food and Drug Administration has announced a new approval pathway for generic biotech drugs as mandated under the PPACA. However, despite growing sales volumes, generic prescriptions generated just 16.5 percent of the pharmaceutical market’s value in 2010. With new drugs entering clinical trials to treat diseases that impact millions of senior citizens, including cancer, Parkinson’s, arthritis, and Alzheimer’s, branded drugs will continue to lead total sales. The PPACA incorporates multiple incentives and mandates for preventive care; to the extent these translate into prescription drug use to delay or ameliorate chronic disease they will have a profound affect on the distribution of health spending in the United States.

The PPACA also contains provisions calling for cost-benefit studies by the government and the industry. An advisory Patient-Centered Outcomes Research Institute (PCORI) will draw greater attention to the cost of prescription drugs relative to health outcomes [34]. For some diseases, outcomes research may drive greater preventive care in the form of prescription drug use and attendant reductions to surgery or other hospital-based treatments. However, for many treatments it will take a decade or more to measure with accuracy gains that accrue from prescription drug use. Even though PCORI lacks regulatory authority, pharmaceutical firms are likely to respond with greater market segmentation for prescription drug therapy by disease subgroups and patient gender, ethnicity, and age. Companies that are able to meet emerging criteria for demonstrating the cost efficiency of their patent-protected drugs will see rising sales, especially as greater access to primary care under the PPACA results in more preventive care through pharmaceuticals.

In China, drug prices have become a topic of concern to policymakers who expect an expanded EDL to drive down prices and increase access to pharmaceuticals. At the same time, the H&FPC allows manufacturers of drugs not on the EDL list, including newly approved medicines, to negotiate prices directly with hospitals. The government thus appears to be seeking a balance between access for patients that pay out of pocket for pharmaceuticals and incentives for firms to invest in research and new drug development. Research-oriented pharmaceutical firms face the challenge of meeting government-set prices for drugs on the EDL while continuing to build markets for their branded offerings. Nevertheless, China’s currently fragmented generics industry and public fears of fake or diluted domestically produced medicines offer significant potential for the growth of branded pharmaceutical sales. Firms can build good will with the government by meeting low price points for pharmaceuticals on the EDL while earning greater margins on higher-priced drugs for other conditions.

The EDL and a more expansive national reimbursement drug list (some 1,700 medicines that may be covered by insurance to some degree) will combine with greater insurance coverage to drive consolidation of the industry. The domestic drug sector will experience competitive pressure to standardize manufacturing, upgrade old facilities, and invest in research and development. However, even the growth of insurance is not changing prevailing norms of out-of-pocket payments for prescriptions. As a result, Chinese doctors and consumers, not insurers or a health outcomes research institute, will be the main targets of industry marketing and medical information. The market is poised for blockbuster drugs, especially treatments for high cholesterol, diabetes, and other chronic diseases. For the industry, competition will involve demonstrating public benefits by meeting requirements for the EDL but also marketing a portfolio of compounds to brand-conscious consumers who pay directly for pharmaceuticals.

## Conclusion

A combination of expanding health insurance coverage, free-market drug pricing policies, and government support for biomedical research will make the United States and China the two largest country markets for drug sales and industry R&D in coming years. The two countries are at fundamentally different stages in economic development and exhibit striking contrasts in health insurance markets and the delivery of care. But policymakers in the United States and China face similar challenges of meeting the needs of large ageing populations and managing tensions between incentives for medical innovation and access to affordable drugs. For their part, multinational pharmaceutical companies are engaged in a complex task when they seek to establish coherent strategies for product innovation, marketing, and sales across their two largest future markets.

The calculations of future pharmaceutical market sizes developed here thus provide a basis for general insights concerning healthcare systems and point to continued tensions concerning drug prices and access. More detailed analyses of future markets for specific disease groups and narrower age demographics are needed to develop targeted policy or business investment recommendations. Nonetheless, the side-by-side analysis of future pharmaceutical markets in the United States and China reveals important similarities in the industry competitive structure as large multinationals dominate the upper end of the market with branded drugs developed through extensive research and testing. Each country also has lessons for the other. China’s EDL establishes a baseline set of affordable medicines that reduces policy tensions concerning access to pharmaceuticals while still, in theory, offering firms rewards for inventing new drugs. Meanwhile, policy changes in the United States will encourage greater preventive care; coupled to new outcomes research, the country may see significant improvements in treatment efficiency that China could emulate in the future.

## Endnotes

^a^Agency for Healthcare Research and Quality, “Prescription medicines expenses per person by source of payment”, http://meps.ahrq.gov, accessed December 2013.

^b^U.S. Centers for Medicare and Medicaid Services, “National health expenditure data”, http://www.cms.gov, accessed December 2013; IMS Health, “Top-line industry data,” http://www.imshealth.com, accessed December 2013.

^c^Figures for the contemporary total pharmaceutical market in China range by source and method of calculation. According to the National Health and Family Planning Commission, total sales in 2012 were RMB 1,186 billion ($188 billion), but this figure includes over-the-counter medicines and TCM drugs; estimates by other sources range between RMB 640 billion ($101 billion) and RMB 422 billion ($67 billion) for allopathic prescription drugs only. Exchange rates throughout the article are based on actual rates of $1 = RMB 6.31 for 2012 and $1 = RMB 6.2 for 2013. For 2015, we project $1 = RMB 5.8, and for 2020, forecast $1 = RMB 5.5. These align to market expectations for future exchange rates and policy statements from the People’s Bank of China.
